# Immunofluorescence Rapid Analysis of Bisphenol A in Water Based on Magnetic Particles and Quantum Dots

**DOI:** 10.3390/s25237328

**Published:** 2025-12-02

**Authors:** Nadezhda A. Taranova, Alisa A. Bulanaya, Anatoly V. Zherdev, Boris B. Dzantiev

**Affiliations:** A.N. Bakh Institute of Biochemistry, Federal Research Center “Fundamentals of Biotechnology” of the Russian Academy of Sciences, Leninsky Prospekt, Building 33, Moscow 119071, Russia; taranovana@inbi.ras.ru (N.A.T.); alisa.bulanaya@yandex.ru (A.A.B.); zherdev@inbi.ras.ru (A.V.Z.)

**Keywords:** bisphenol A, magnetic particles, quantum dots, immunochromatographic analysis, concentration

## Abstract

**Highlights:**

**What are the main findings?**

- Magnetic particles and quantum dots are used as an enhancing component, which were able to reduce the detection limit by 20 times compared to the use of magnetic particles alone.
- The use of magnetic preconcentration made it possible to further reduce the detection limit by 100 times.

**What is the main implication of the main findings?**

- The article is devoted to the development of a highly sensitive immunochromatographic assay for the determination of bisphenol A in natural water.

**Abstract:**

Bisphenol A (BPA) is widely used as a hardener in plastics production and its release and circulation in ecosystems lead to negatively impacts the human endocrine system. Therefore, there is a need for simple and efficient BPA monitoring tools. This paper presents a combination of two approaches for this purpose: the use of magnetic particles (MPs) as antibody carriers and immunochromatographic test strips based on quantum dots (QDs) for recording labeled immune complexes. Initially, free antigen binds to the MP-specific antibody conjugate, concentrating the sample to a final volume of 200 µL. A competitive interaction then occurs in the analytical zone of the test strip with immersion in a QDs solution. The visual detection limit of BPA was 2.7 μg/mL, the instrumental detection limit was 0.03 μg/mL, and the working range of quantification was 0.3–100 μg/mL (reproducibility was 7.7%, R^2^ = 0.985). Concentration using MP reduces the detection limit by 100-fold (0.3 ng/mL). The developed test was used for qualitative control of the presence and quantitative determination of BPA content in samples of drinking and natural water (the detection rate is in the range of 94–110%).

## 1. Introduction

In the modern world, special attention is paid to the quality of life and human health, as well as the environmental health of the environment. BPA is known to have a negative impact on the endocrine system, due to its structural similarity to hormones such as estradiol [1,2]. By binding to estrogen receptors, BPA disrupts natural hormonal regulation [3]. Even in very low doses, BPA has been shown to affect thyroid function, endocrine activity of the pancreas, functioning of the reproductive organs and the immune system [4,5]. Recent research data indicate that exposure to BPA on the body is accompanied by hepatotoxicity, neurotoxicity, and increases the likelihood of developing diabetes and cardiovascular diseases [6].

Given these negative impacts on human health, governments in Russia, the United States, the European Union, Japan, China, and other countries have begun setting maximum permissible concentrations of BPA in food, air, and drinking water. The global regulatory framework for BPA varies widely [7].

The detection of bisphenol A (4,4’-(propane-2,2-diyl)diphenol, BPA) in the environment and food products has become a highly sought-after task [8,9]. BPA is used primarily as a monomer for the synthesis of polymeric materials for household and technical purposes, including polycarbonate, polysulfone, polyester, and epoxy resins [10,11]. Thermal processing can lead to the release of BPA from such materials into food. It is estimated that more than 100 tons of BPA are released into the environment each year [12]. Bisphenol A has been detected in rivers, groundwater, surface water, and seas worldwide [2,8].

Despite the diversity of existing methods for detecting BPA [8,9,13], liquid and gas chromatography coupled with different types of detectors are predominantly used for these purposes [14,15]. Thus, UV detectors, fluorescence detectors and detectors based on mass spectrometry (MS) are used to detect BPA based on gas chromatography (GC) [16,17]. A number of highly sensitive methods for the quantitative determination of BPA, combining LC and MS, are described [7,10]. For methods based on the use of capillary electrophoresis, rapid results are obtained and low consumption of reagents and samples is noted [18,19]. However, the implementation of all these methods requires complex, expensive equipment and highly qualified personnel, which limits their use.

Recently, bioanalytical methods based on the binding of detectable compounds to selective receptor molecules have been widely used to solve various problems. These methods are characterized by high sensitivity, simple, rapid implementation, and cost-effectiveness. A number of bioanalytical methods have been developed for the detection of BPA [20,21,22,23,24], using antibodies, molecularly imprinted polymers, and aptamers as receptors. The choice of a specific receptor determines the choice of the format of the bioanalytical system, the diversity of which includes microfluidic, flow (columns, test strips), disk, agglutination, and enzyme systems [25,26,27].

Lateral flow immunoassay (LFIA) has become one of the most successful analytical platforms for decentralized testing that does not require supporting infrastructure [28,29,30]. The popularity of LFIA is due to its advantages: ease of implementation, high stability of test systems during storage, rapid (5–15 min) obtaining of results, the possibility of using it as a semi-quantitative and quantitative analytical method [31].

Immunochromatographic test systems for the detection of BPA are described, in which the use of markers traditional for LFIA—gold nanoparticles or latex spheres—makes it possible to achieve detection limits of up to 10 ng/mL [32,33]. For more highly sensitive analysis, alternative markers and non-optical methods of their registration, various methods of signal amplification or complex sample preparation with BFA concentration are proposed [34,35].

In our work, we propose to implement a two-stage immunochromatographic analysis of BPA in natural and drinking water, allowing the detection of low concentrations of the analyte by pre-concentration from a large sample volume using an immunosorbent based on MPs with subsequent detection using QDs. The primary goal of using magnetic particles was to ensure sample concentration, which can be accomplished more easily and quickly with magnetic particles than with alternative methods. Furthermore, the use of two particle types in the proposed format provides a means of marker aggregation and signal amplification. The article proposes for the first time the independent use of individual magnetic particles and quantum dots. The use of combined preparations (magnetic quantum dots with lower fluorescence) requires a more complex synthesis process.

## 2. Materials and Methods

**Reagents and materials.** Iron (II) chloride (FeCl_2_), iron (III) chloride (FeCl_3_), hydrochloric acid, sodium hydroxide, tris, dimethyl sulfoxide (DMSO), bisphenol A (BPA), 4,4-bis(hydroxyphenyl)valeric acid (BVK), soybean trypsin inhibitor (STI), N-hydroxysulfosuccinimide, 1-ethyl-3-(3′-dimethylaminopropyl)carbodiimide, hydrogen tetrachloroaurate (HAuCl4), sodium citrate, nonionic detergent Tween-20, sodium azide, trimethyl ammonium chloride (TMACl)—manufactured by Sigma Aldrich (St. Louis, MO, USA). Water-soluble carboxylated polymer-coated CdSe/ZnS quantum dots (emission peak at 625 nm) from Invitrogen (Waltham, MA, USA) were used in this study. Bovine serum albumin (BSA), sodium chloride, potassium hydroxide, and potassium dihydrogen phosphate were purchased from Khimmed (Moscow, Russia). All auxiliary reagents (salts, acids, alkalis, and organic solvents) were of analytical or chemical grade.

Rabbit antisera (aAs) against BPA were previously obtained [36] in the vivarium of the Federal Research Center of Biotechnology of the Russian Academy of Sciences. Goat anti-rabbit IgG antibodies (Ab) were purchased from HyTest (Moscow, Russia).

All aqueous solutions were prepared using deionized water with a specific resistance at 25 °C of at least 18.2 MΩ cm, using the KH.3LAB-20S ultrapure water production system from MT-Master (Moscow, Russia).

**Synthesis of magnetic particles.** Magnetic particles were synthesized according to the procedure described in [37]. 3.42 g FeCl_2_, 9.19 g FeCl_3_ and 100 mL 2 M HCl were added to 20 mL of water. Then 300 mL 5 M NaOH were added under vigorous stirring (860 rpm) using an Ekros-8300 overhead stirrer (Ekros, Sant-Petersburg, Russia). The reaction mixture was stirred for 30 min. The obtained particles were precipitated using a neodymium magnet (diameter 6 cm, height 3 cm, adhesion force 70 kg) and transferred to bidistilled water. To stabilize the surface, the magnetic particles were mixed with 50 mL 0.1 M TMACl. The synthesized preparation was stored at 4 °C.

**Magnetic particle characterization.** For transmission electron microscopy (TEM), magnetic particle samples were applied to 300-mesh grids (Pelco International, Fresno, CA, USA) coated with a polyvinyl formalin film. Images were acquired using a JEM CX-100 microscope (Jeol, Akishima, Tokyo, Japan) at 80 kV and analyzed using Image Tool 2.0 software (University of Texas Health Science Center, Houston, TX, USA).

The hydrodynamic size and ζ-potential of magnetic particles were determined using a Zetasizer Nano instrument (Malvern Pananlytical, Worcestershire, UK). Dynamic light scattering was recorded at 25 °C for 10 s at a scattering angle of 173°.

**Preparation of BVK conjugates with protein.** With few changes, the activated ester technique described in [38]. 200 μL of deionized water was used to dissolve 2.4 mg (21 μmol) of N-hydroxysulfosuccinimide and 4.1 mg (26 μmol) of 1-ethyl-3-(3′-dimethylaminopropyl)carbodiimide. BVK (4.88 mg, corresponding to 17 μmol) was dissolved in 200 μL of DMSO and added to the activator solution. The resulting mixture was incubated at room temperature for 24 h with stirring. 10 mg of STI were dissolved in 1 mL of phosphate-buffered saline (PBS) containing 10 mM potassium dihydrogen phosphate and 20 mM sodium chloride, pH 7.4, and 200 μL of the obtained activated BVK derivative were added. Thus, the BVK:STI molar ratios ranged from 15:1 to 70:1. The DMSO content in the reaction mixture was 14.3%. Following five hours of vigorous stirring at ambient temperature, the reaction mixture was centrifuged using Amicon tubes with a molecular weight cutoff of 10 kDa (Merck, Darmstadt, Germany) for ten minutes at 10,000× *g* to dialyze the resultant conjugates against PBS. The conjugates were divided into aliquots and stored at −20 °C.

The absorption spectra of the obtained conjugates were recorded in the wavelength range of 220–700 nm using a Libra S80 spectrophotometer (Biochrom, Cambridge, UK).

**Isolation of the immunoglobulin G fraction [39].** 1 mL of the antiserum was mixed with 270 mg of ammonium sulfate [36], and the mixture was agitated for 30 min at room temperature (RT). After centrifuging the mixture for 10 min at 9500× *g* to precipitate it, the supernatant was removed. For 24 h, the precipitate was dialyzed against 2 L of FBS after being dissolved in 1 milliliter of FBS. At −20 °C, the resultant polyclonal anti-body (PAb) preparation was kept.

**Synthesis of a magnetic particle–antibody conjugate (MP-PAb).** The magnetic particle surface was employed to adsorb polyclonal antibodies against BPA (PAb). First, the magnetic particles were moved to pH 8.0 Tris-buffer. 200 μL of BSA was combined with 1 mL of colloidal magnetic particle solution (3 mg/mL) and 13.3 μL of antibodies (10.5 mg/mL), which were then continuously stirred for 45 min. A magnet was used to precipitate the resultant conjugate, and Tris-buffer (pH 7.5) was used for washing

**Synthesis of a conjugate of QDs with anti-species antibodies (QD-Ab).** The conjugation method presented in [40] was used as a basis. A tenfold excess (mol/mol) of antibodies relative to quantum dots was used for the synthesis of the conjugate. For this purpose, 25 μL of a QD solution in a borate buffer solution (pH 8.0, BB) with a concentration of 8 μM were added to 300 μL of an antibody solution in PBS with a concentration of 1 mg/mL. Then, 50 μL of EDC and sulfo-NHS activator solutions with an initial concentration of 1.6∙10^−3^ M were sequentially added to the mixture. Weighed portions of the activators were dissolved in deionized water immediately before use. The molar ratio of QDs: activator was 1:400. Conjugate synthesis was performed with constant stirring on a shaker (Intelli-Mixer RM-2, ELMI, Sant-Petersburg, Russia) for 90 min at room temperature in the dark. Concentration was then performed with simultaneous dialysis against BB to remove excess activators using Amicon Ultracel 100K microcentrifuge tubes at 10,000× *g* for 15 min. After five centrifugations, the 10-fold concentrated conjugate was collected. The resulting conjugate was stored at 4 °C.

**Manufacturing of lateral flow immunoassay test strips.** Mdi Easypack membrane kits (nitrocellulose working membranes and an absorbent membrane) from Advanced Micro-devices (Ambala Cantt, Haryana, India) and Millipore (Darmstadt, Germany), which vary in pore size, were used to produce immunochromatographic test systems.

The SIT-BVK conjugate (1 mg/mL in PB, solution flow rate 1.2 μL per 1 cm of strip length) and anti-species polyclonal antibodies (1 mg/mL in PB, 1.2 μL per 1 cm) were applied using a dispenser (Imagene Technology, Lebanon, NH, USA) to create the test and control zones (T.Z. and C.Z., respectively) on a sheet with a working nitrocellulose membrane (length 24 cm, sheet width 8 cm, and working membrane width 2.5 cm). At 37 °C, the membranes were dried for two hours. A guillotine cutter (Shanghai Kinbio Tech. Co., Shanghai, China) was then used to build and cut the multimembrane composite comprising the working and ab-sorbent (cellulose) membranes into test strips that were 3.5 mm wide. The test strips were kept at room temperature with silica gel present, enclosed in foil wrapping.

**Conducting lateral flow immunoassay (LFIA).** At room temperature, the MP-antibody conjugate (3 mg/mL) was added to a 0.1 mL test sample. The edge of the absorbent membrane of the test strip was immersed in the resulting mixture for 15 min. The test strip was then placed in the QD-Ab conjugate solution for 10 min. Upon completion of the test, the test strips were removed and digital images were acquired using a UV-photocamera. TotalLAB TL120 software (Nonlinear Dynamics, Manchester, UK) was used to quantify the staining intensity of the test and control zones.

**Lateral flow immunoassay with immunomagnetic preconcentration.** Test samples (1 to 50 mL) were mixed with the MP-antibody conjugate (3 mg/mL) and incubated for 5 min at room temperature. The MPs and associated substances were then separated using a magnetic field. After removing the magnet, the precipitate was resuspended in 0.1 mL of PBS, and immunochromatography was performed as described above. The dependences of the recorded color intensity (y) on the analyte concentration (x) were approximated by a linear function using OriginPro 2021 software (OriginLab, Northampton, MA, USA).

The minimum analyte concentration that resulted in the disappearance of the analytical zone was considered the visual LOD. The instrumental LOD corresponded to the analyte concentration at which the color intensity of the analytical zone decreased by three times the standard deviation of the background color (i.e., for samples containing no analyte).

## 3. Results

**Figure 1:**
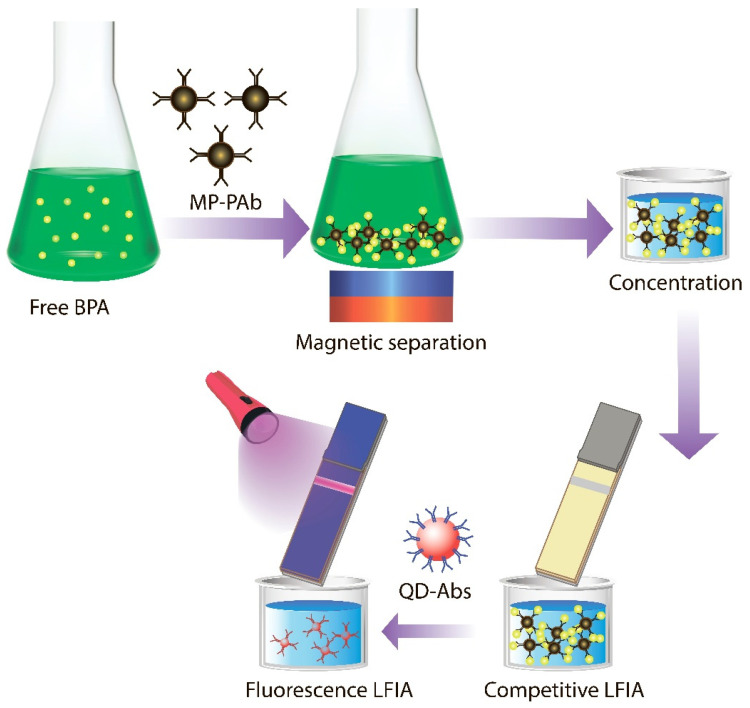
Scheme of conducting LFIA with amplification of the analytical signal and preliminary concentration.

**Synthesis and characterization of magnetic particles** A variant of the LFIA with sequential detection of BPA in large-volume samples is proposed, based on the use of MPs as a preconcentrating agent and subsequent detection using high-fluorescence QDs. The proposed assay scheme is shown in Figure 1.

Since BPA does not contain active groups for conjugation, its derivative, 4,4-bis(hydroxyphenyl)valeric acid (BVA), containing a carboxyl group, was used. BVA was covalently linked to soybean trypsin inhibitor using activators [36].

For the synthesis of MPs, the most widely used transformation of soluble Fe^2+^ and Fe^3+^ salts in the presence of a base with subsequent formation of an insoluble mixed oxide Fe_3_O_4_ was used:

Fe^2+^ + 2Fe^3+^ + 8OH^−^ → Fe(OH)_2_ + 2Fe(OH)_3_ → Fe_3_O_4_ + 4H_2_O

**Figure 2:**
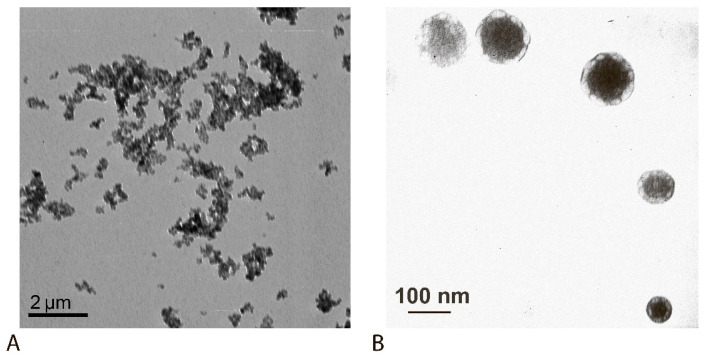
TEM characterization of the obtained MP preparation: wide-field image showing aggregates (**A**) and its enlarged part with individual particles (**B**).

Sodium hydroxide was used as a base, added to a solution of iron salts with vigorous stirring. The amount of sodium hydroxide added was monitored visually until a stable black color formed. After purification of the synthesized magnetic particles from reaction products, the particles were stabilized by adding TMACl, which forms a positively charged layer on the surface of the magnetic particles, partially preventing their aggregation (Figure 2A).

**Figure 3:**
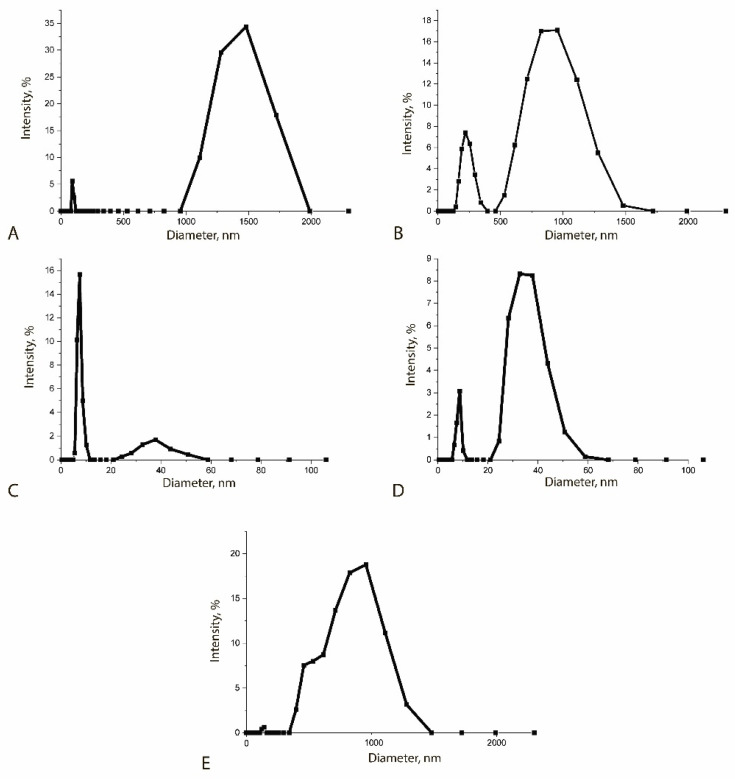
Distribution of hydrodynamic diameter of: MPs (**A**), MP-PAb (**B**), QDs (**C**), QD-Ab (**D**) and MP-PAb–QD-Ab (**E**).

The MP sampels were characterized by dynamic light scattering (Figure 3). A hydrodynamic study revealed that unmodified MPs in solution exist predominantly in the form of aggregates (1500 ± 200 nm) formed under the influence of their own magnetic field (Figure 3A).

**Figure 4:**
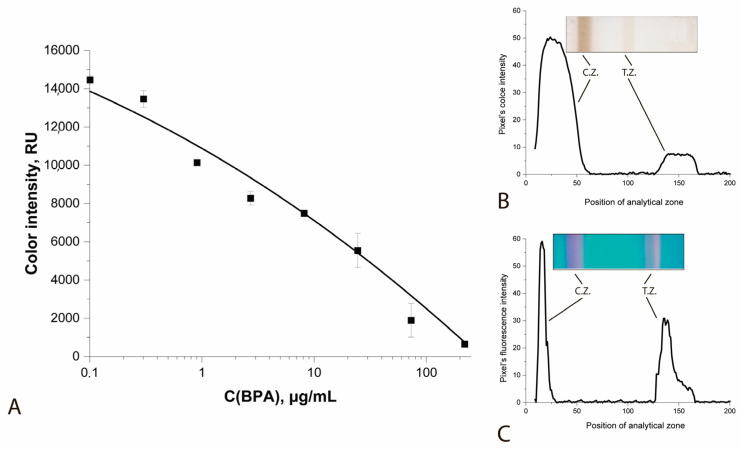
Calibration curve of the LFIA BPA in a standard format (**A**), appearance of the test strip and color distribution profile when using only the MP-PAb conjugate (**B**) and with enhancement of QD-Ab (**C**) (C.Z.—control zone, T.Z.—test zone).

**LFIA with magnetic particle–antibody conjugate (MP-PAb).** The conjugate of magnetic particle–antibody conjugate (MP-PAb) was used in the standard format of competitive BPA LFIA (Figure 4A,B). This scheme was characterized by very low staining intensities of the analytical and control zones and a high detection limit (LOD) of 663 ng/mL.

**Figure 5:**
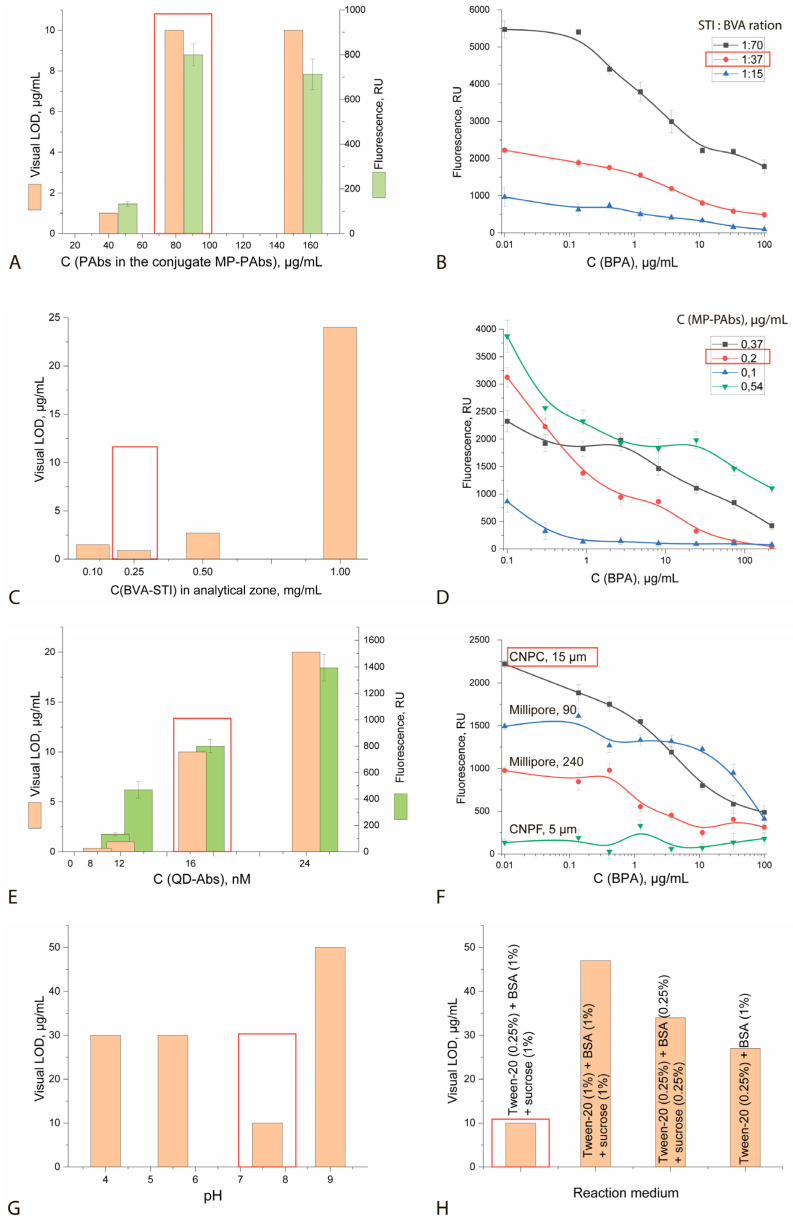
Results of experiments on selecting the parameters of the ICA of BPA with a modified fluorescence signal: (**A**)—concentration of PAb in the MP-PAb conjugate; (**B**)—location of the SIT:BVK; (**C**)—concentration of the SIT-BVK conjugate transferred to the analytical zone; (**D**)—concentration of the MP-PAb conjugate; (**E**)—concentration of the QD-Ab conjugate; (**F**)—type of the working membrane and its pore size; (**G**)—pH of the reaction medium; (**H**)—composition of the reaction medium. Red box shows the choosen parameter.

The selection of the test system parameters was based on sufficient fluorescence intensity of the analytical zone and minimal visual diffraction. Thus, with a decrease in the PAb content in the MP-PAb conjugate, the fluorescence intensity of the analytical zone drops sharply, which reduces the reliability of the results obtained (Figure 5A). With an increase in the SIT:BVK ratio during conjugate synthesis to 1:70, the visual diffraction increases 5-fold, and with a decrease to 1:15, it drops to 1 μg/mL with a simultaneous decrease in the fluorescence intensity of the analytical zone (i.e., a deterioration in the reliability of the results) (Figure 5B). An increase in the STI-BVK conjugate concentration in the analytical zone leads to a 10-fold increase in the visual diffraction (Figure 5C). An increase in the concentration of the MP-PAb conjugate in the reaction mixture leads to a 3-fold increase in the visual LOD, while a decrease in the concentration leads to a decrease in the reliability of the results (Figure 5D). Varying the concentration of the QD-Ab conjugate in the reaction mixture resulted in a direct relationship between the visual LOD and the fluorescence intensity of the analytical zone (Figure 5E). A decrease in the pore size of the working membrane resulted in a decrease in the fluorescence intensity of the analytical zone and an increase in the visual LOD (Figure 5F). A shift in the pH of the reaction mixture toward lower values (<7.0) resulted in the dissolution of some of the MP, which resulted in the appearance of free PAbs in the reaction medium and, therefore, an increase in the visual LOD (Figure 5G). It was also shown that, for highly sensitive detection of BPA, the blocking reagents in the reaction mixture should include a detergent (Tween-20), BSA, and sucrose (Figure 5H).

**Figure 6:**
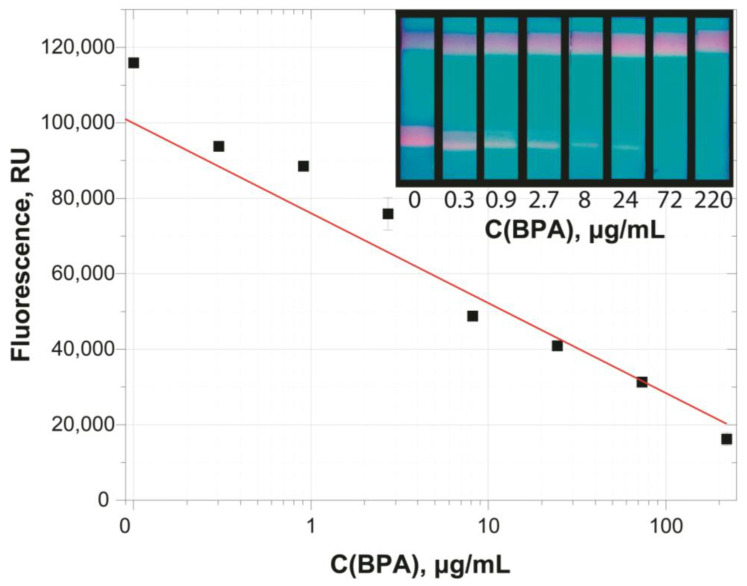
Calibration curve for the determination of BPA and the appearance of test strips.

Based on the selected parameters, a test system for detecting BPA in water samples was developed. Figure 6 shows the calibration curve and the appearance of the test strips.

The concentration dependence (Figure 6) is approximated of the working range in semi-logarithmic coordinates by the dependence (R^2^ = 0.985):

y = 83,356 − 29,961 × x.

To reduce the detection limit, it was decided to concentrate BPA using a magnetic particle conjugate. To evaluate the feasibility of this approach, a BPA concentration of 0.1 μg/mL was chosen, as the observed coloration of the analytical zone in the LFIA variant without concentration is virtually the same as for negative samples.

**Analysis of water samples.** Experiments were conducted for sample volumes of 1, 5, and 10 mL with resuspension in 100 μL, which corresponds to a concentration of 10, 50, and 100 times. Data on the color intensity of the analytical zones are presented in Table 1.

The data presented in the table show that increasing the sample volume with the same analyte concentration leads to a decrease in the color intensity of the analytical zone, indicating antigen detection. Table 2 presents data on BPA detection in LFIA with concentration.

Three water samples (two well water and one drinking water) contaminated with BPA were analyzed using the developed immunochromatographic method. The test results are presented in Table 3.

According to the data obtained, the detection rate is in the range of 94–110%. Thus, the developed LFIA is suitable for the quantitative and qualitative determination of BPA content.

## 4. Discussion

**Choice of the analytical system design.** A promising solution that overcomes the limitations of classical sample preparation methods is the use of dispersions of magnetic particles (MPs) with antibodies immobilized on their surface [41,42]. Magnetic immunosorbents enable the sequential implementation of: (1) analyte binding (rapid due to interactions throughout the sample volume), (2) separation of the resulting MP–analyte complexes from sample components upon application of an external magnetic field, and (3) resuspension of these complexes in a small volume after removal of the magnet. This sample preparation enables the rapid and rapid increase in analyte content in the final test sample and the elimination of components of the original sample that could potentially interfere with specific interactions and signal generation.

However, MPs have low staining intensity, which significantly reduces the sensitivity of immunochromatographic test systems. An alternative solution proposed is to combine the magnetic properties of MPs with the unique spectrophotometric properties of known markers, such as quantum dots (QDs).

Fluorescence LFIA offer advantages over traditional approaches in terms of sensitivity because they produce a more intense band on test and control lines. QDs are tiny semiconductor nanocrystals with diameters ranging from 2 to 10 nanometers [43,44]. QDs exhibit unique electronic properties that are intermediate between those of bulk semiconductors and discrete molecules, partly due to their high surface-to-volume ratio. The most notable result is fluorescence, in which nanocrystals emit various colors determined by particle size [45]. The use of QD as a fluorescent marker allows to reduce the detection limit by 10–20 times relative to colloidal gold [46,47].

A number of studies have described the use of QDs containing a subunit with magnetic properties as a core [38,48]. However, such drugs have lower fluorescence levels and require complex synthesis methods. Combined QDs have found widespread application in bioimaging [49,50].

Combining the unique properties of MPs and QDs will reduce the analyte detection limit in two ways: (a) by concentrating from a large sample volume; (b) by enabling more sensitive fluorescence detection. Stepwise application of the markers will allow their use in immunochromatographic analysis due to the preservation of small particle sizes (up to 500 nm).

To improve the performance of the LFIA, we implemented signal amplification: after the MP-Ab conjugate passed through the test strip, a conjugate of quantum dots with anti-species (goat anti-rabbit) antibodies (QD-Ab) was passed through (Figure 1). QDs exhibit highly stable fluorescence. Incorporating QDs into complexes with MPs significantly increases the staining intensity of the test strip’s analytical zone.

**Characteristics of the obtained reagents.** According to the obtained TEM data, the diameter of the synthesized individual particles was 80 ± 25 nm (Figure 2B), while the size of the aggregates reached 1.5 µm (Figure 2A). MPs have their own magnetic field, which leads to the formation of aggregates from unmodified MPs [51,52]. A hydrodynamic study revealed that unmodified MPs in solution exist predominantly in the form of aggregates (1500 ± 200 nm) formed under the influence of their own magnetic field (Figure 3A). The MP preparation also contains a fraction of particles with a diameter of 88 ± 10 nm. The synthesized MPs were adsorption-modified with polyclonal antibodies isolated from rabbit serum. Stabilization of MPs with proteins reduced the proportion of large aggregates: particle sizes reached 250 ± 15 nm and 855 ± 20 nm (Figure 3B).

To assess the formation of the MP-PAb–QD-Ab complex, measurements of individual QDs, their antibody conjugates, and the complexes themselves were also conducted (Figure 3). The sizes of the particles and their complexes are presented in Table 4.

To achieve minimal LOD, a series of LFIA optimizations were performed, including selection of the concentration of immobilized specific polyclonal antibodies, the hapten-to-protein ratio in the BVK-SIT conjugate, the concentration of the BVK-SIT conjugate applied to the assay zone, the concentrations of the MP-PAb and QD-Ab conjugates, the working membrane, and the composition of the assay medium (pH, detergent content, and blocking reagents). The optimal configuration of the test system and reaction mixture is summarized in Table 5.

**Analytical parameters of the developed assay.** The visual detection limit of BPA was 2.7 μg/mL, the instrumental limit was 0.03 μg/mL, and the working range of detectable concentrations was 0.3–100 μg/mL. The reproducibility of the obtained results was 7–10%, relative standard deviation was 1–8%, and the data convergence ranges from 8.5 to 12%. No non-specific cross-reactions were observed. Cross-reactivity of the developed test system with the listed plasticizers was demonstrated to be absent. The selectivity of the developed test system depends on the specificity of the polyclonal antibodies, which were previously characterized using nonylphenol, dibutyl phthalate, and diisobutyl phthalate [53].

**Comparison with earlier developments.** Table 6 presents the existing methods for detecting BPA. Most instrumental methods (HPLC, GC-MS, electrophoresis) require complex, specialized equipment, making them unsuitable for external testing. Rapid methods require the use of complex receptors. Analysis requires complex sample preparation (antigen derivation).

Instrumental analytical methods, such as (HPLC, GC-MS, electrophoresis), are characterized by a wide range of analysis times. For example, HPLC takes 10 min in sequential analysis of samples, while electrophoresis takes 60 min. The sample volume required for analysis depends on the number of replicates and typically does not exceed 1 mL for direct measurements (without sample preparation). However, when concentrating the analyte, a significantly larger sample volume is required: extraction with evaporation in chromatography or simple and rapid magnetic concentration. The proposed method for determining BPA is not inferior in sensitivity to existing LFIA test systems. Taking into account the possibility of concentrating the antigen from a large sample volume, these test systems can be used to analyze natural water.

## 5. Conclusions

We proposes to implement a two-stage immunochromatographic analysis of BPA in natural and drinking water, allowing the detection of low concentrations of the analyte by pre-concentration from a large sample volume using an immunosorbent based on MPs with subsequent detection using QDs. The visual detection limit of BPA was 2.7 μg/mL, the instrumental detection limit was 0.03 μg/mL, and the working range of quantification was 0.3–100 μg/mL. Assay time was 20 min. The effectiveness of using a conjugated MPs for analyte preconcentration was demonstrated, allowing the detection limit to be reduced by approximately 100-fold. The developed lateral flow immunoassay (LFIA) is suitable for qualitative control of the presence and quantitative determination of BPA content in samples of drinking and natural water (the detection rate is in the range of 94–110%).

The method utilizes fluorescence detection and therefore requires additional means for excitation. However, by using quantum dots as fluorophores, this excitation with broadband UV radiation can be achieved using mass-produced household devices. This study demonstrates only the fundamental feasibility of using magnetic preconcentration when working with water samples and the resulting gains in sensitivity. Therefore, a promising direction for further development is a detailed study of preconcentration processes to select the most effective conditions, an evaluation of the effectiveness of the developed method for monitoring bisphenol in a wide range of matrices, and the expansion of the application of the developed approach, combining magnetic and fluorescent highly dispersed markers, to the immunochromatography of other analytes.

## Figures and Tables

**Table 1:** Color intensity of analytical zones of test strips during LFIA with immunomagnetic preconcentration from different volumes.

	Color Intensity of Analytical Zones, Relative Units *			
Initial Concentration of BPA, μg/mL	Without Concentration	Concentration from 1 mL	Concentration from 5 mL	Concentration from 10 mL
0.01	123,700 ± 1700	99,780 ± 1200	83,420 ± 990	75,970 ± 840
0.1	99,990 ± 950	75,250 ± 1090	59,090 ± 1200	52,160 ± 1170
1	76,380 ± 780	50,900 ± 1300	35,500 ± 790	27,700 ± 690

*—the area of the peak of the test strip color distribution profile located in the analytical zone (Figure 4B,C).

**Table 2:** Results of BPA LFIA with magnetic preconcentration.

	BPA Concentration, µg/mL			
Initial Concentration of BPA, μg/mL	Without Concentration	Concentration from 1 mL	Concentration from 5 mL	Concentration from 10 mL
0.01	0.011 ± 0.003	0.099 ± 0.005	0.49 ± 0.04	0.9 ± 0.1
0.1	0.098 ± 0.007	1.1 ± 0.1	11.4 ± 0.9	10 ± 1
1	0.98 ± 0.10	11.2 ± 0.7	50 ± 2	107 ± 9

**Table 3:** ICA testing of water samples contaminated with LFIA (*n* = 3).

Sample Type	Added, µg/mL	Detected, µg/mL	Recovery, %
Drink water	3	3.2 ± 0.5	107 ± 8
	1	1.1 ± 0.3	110 ± 9
	0.3	0.29 ± 0.03	97 ± 5
Well 1	3	3.3 ± 0.4	110 ± 8
	1	0.94 ± 0.08	94 ± 7
	0.3	0.32 ± 0.04	107 ± 6
Well 2	3	3.1 ± 0.6	103 ± 5
	1	1.04 ± 0.05	104 ± 9
	0.3	0.30 ± 0.10	100 ± 5

**Table 4:** Sizes of particles and their conjugates and complexes (DLS).

Sample	Size, nm
MPs	88 ± 10 and 1500 ± 200
MP-PAb	250 ± 15 and 855 ± 20
QDs	8 ± 3
QD-Ab	47 ± 18
MP-PAb–QD-Ab	838 ± 95

**Table 5:** Optimal configuration of the test system and reaction mixture for ICA of BPA with signal amplification.

Parameters	Value
PAb concentration in the MP-PAT conjugate	80 µg/mL
SIT:BVK ratio during conjugate synthesis	1:37
SIT-BVK conjugate concentration applied to the analytical zone	0.25 mg/mL
MP-PAb conjugate concentration	0.2 mg/mL
QD-Ab conjugate concentration	16 nM
Working nitrocellulose membrane	Type CNPC (high protein binding), average pore size: 15 μm
Reaction medium pH	7.6
Reaction medium composition	Tween-20 (0.25%) + BSA (1%) + sucrose (1%)

**Table 6:** Realized systems for BPA detection.

Method	Receptor	Sample Preparation	LOD	Object	Ref.
Amperometric detection	Without receptor	Standard sample	1.38 × 10^−7^ M (31.5 ng/mL)	PC water bottle	[54]
Fluorescence sol–gel biochip	Aptamer	Standard sample	1 pM (0.23 fg/mL)	-	[55]
Chemiluminescent system	Aptamer	Standard sample	1 μM (0.23 μg/mL)	Water	[56]
HPLC	Without receptor	Derivatization of the extract	0.71 pg/mL	-	[57]
GC–MS	Without receptor	Derivatization of the extract	2.0 ng/g	Honey	[58]
Capillary electrophoresis	Without receptor	Microextraction	0.6 mg/mL	Tap water, lake water and seawater samples	[59]
LFIA with QDs	Antibody	-	10 ng/mL	Distilled drinks	[60]
LFIA with latex particles	Antibody	Extraction	0.14 ng/mL	coated papers	[34]
LFIA with GNPs	Antibody	-	0.67 ng/mL	River water	[53]
Our work	Antibody	-	0.3 ng/mL (with preconcentration)	Water	-

## Data Availability

The original contributions presented in this study are included in the article. Further inquiries can be directed to the corresponding author.
